# The Next Generation of Sustainable Food Packaging to Preserve Our Environment in a Circular Economy Context

**DOI:** 10.3389/fnut.2018.00121

**Published:** 2018-12-04

**Authors:** Valérie Guillard, Sébastien Gaucel, Claudio Fornaciari, Hélène Angellier-Coussy, Patrice Buche, Nathalie Gontard

**Affiliations:** ^1^UMR IATE, University of Montpellier, INRA, SupAgro, CIRAD, Montpellier, France; ^2^Coopbox Group S.p.A., Bibbiano, Italy

**Keywords:** food packaging, sustainability, biodegradable, bio-sourced, waste-based

## Abstract

Packaging is an essential element of response to address key challenges of sustainable food consumption on the international scene, which is clearly about minimizing the environmental footprint of packed food. An innovative sustainable packaging aims to address food waste and loss reduction by preserving food quality, as well as food safety issues by preventing food-borne diseases and food chemical contamination. Moreover, it must address the long-term crucial issue of environmentally persistent plastic waste accumulation as well as the saving of oil and food material resources. This paper reviews the major challenges that food packaging must tackle in the near future in order to enter the virtuous loop of circular bio-economy. Some solutions are proposed to address pressing international stakes in terms of food and plastic waste reduction and end-of-life issues of persistent materials. Among potential solutions, production of microbial biodegradable polymers from agro-food waste residues seems a promising route to create an innovative, more resilient, and productive waste-based food packaging economy by decoupling the food packaging industry from fossil feed stocks and permitting nutrients to return to the soil. To respond to the lack of tools and approach to properly design and adapt food packaging to food needs, mathematical simulation, based on modeling of mass transfer and reactions into food/packaging systems are promising tools. The next generation of such modeling and tools should help the food packaging sector to validate usage benefit of new packaging solutions and chose, in a fair and transparent way, the best packaging solution to contribute to the overall decrease of food losses and persistent plastic accumulation.

## Introduction

Around 100 million tons of foods are wasted annually in the EU, nearly 30% of the agri-food supply chain (1), which leads to huge environmental impacts (high carbon footprint and blue water footprint, vain land use, etc.) (2, 3). Food waste should rise to over 200 million tons by 2050 while an increase of 50% in food supplies will be needed globally (4, 5). Even if the relation between shelf-life and food waste is not straightforward, a large part of food wastage is related to the short shelf-life of a lot of fresh produce inherent to its biological origin. Moreover, inaccuracies in, or misunderstanding of, food date labels are estimated to cause over 20% of the avoidable disposal of still-edible food (6).

Recently, packaging was identified as an essential element to address the key challenge of sustainable food consumption and is gaining interest among scientists (7, 8). Packaging is a central element to food quality preservation by mainly, controlling gas and vapor exchanges with the external atmosphere, contributing to preserving food quality during storage, preventing food safety issues (prevention of food-borne diseases and food chemical contamination) and extending food shelf-life. Significant benefits are expected in terms of reduction of food waste thanks to shelf life extension (9, 10), especially by using a well dimensioned packaging material, adapted to food needs in term of preservation (8, 11). However, packaging is usually wrongly considered as an additional economic and environmental cost rather than an added value for waste reduction. Moreover, primary packaging^1^ is, currently, not always well adapted to the food needs and therefore does not efficiently and sufficiently contribute to maintain the shelf life of the food (9, 10, 12).

When a food product is thrown away, the packaging is also discarded leading to an additional environmental burden. In our plastic based economy, packaging materials are principally oil-based. Plastic world production increased by 4.2% between 2015 and 2016 to reach 335 million tons. 23 million tons of plastic packaging are produced each year in Europe (92 million tons expected in 2050)^2^. After an exclusively single and very short use inherent as food packaging, 40% ends up in landfill corresponding to 9 million tons of plastic packaging waste that is fated to accumulate in soils. 32% leak out of collecting and sorting systems and finally end in the soil and ocean as well (13, 14). This marine and soil litter first degrades into micro- and then into nano-sized particles that could thus easily penetrate into living organisms such as fish and then be fed up the food chain, all the way to humans with dramatic deleterious long-term adverse effects (15). If production and use continue within the current linear framework, and if nothing is done by 2050 there may be more plastic than fish in the ocean, by weight (13).

To tackle issues related to oil-based packaging, a lot of attention has been paid to raw materials to replace non-renewable oil resources. However, currently marketed bio-sourced bio-plastic (such as Bio-PE, PLA, and more) use food resources such as corn or cane sugar. They contribute to increase food security concerns and pressure on agricultural land (16). Moreover, most of these bio-sourced bio-plastics are not biodegradable nor home-compostable (bio-PE, bio-PET) or are fit only for industrial composting (PLA) which contributes to complicating the waste management: separate collecting and sorting of these materials are thus needed (17, 18). The term “bio” itself appears confusing for consumers, referring on one hand to the nature of resources and on the other hand to material end-of-life (biodegradability). Finally, commercially available eco-efficient packaging solutions are facing difficulty in being considered convincing “sustainable packaging” because their economic and environmental “cost vs. benefit” balance is not obviously and simply demonstrated, or even controversial, for most stakeholders who request trust to be restored and existing green washing suspicion to be lifted.

In this context, it appears that food and packaging waste reduction means more rather than less packaging, or oil-based resources substitution by renewable resources. In addition to mitigating the negative burden of packaging resources and packaging waste management, a sustainable food packaging also increases its positive usage benefit, which is the reduction of food losses and waste. This is achieved by primarily fitting the food requirements to preserve food quality and safety on the whole supply chain and mainly at distribution and consumption stages. Considering the product and its packaging as a complete system is thus primordial to optimize the sustainability of food/packaging systems as a whole.

This paper aims to demonstrate how packaging could be a key element of sustainable food consumption by simultaneously decreasing food waste and losses and the burden on resources and packaging waste management. In a first part, the primary fundamental role of food packaging will be first recalled, then, in a second part, the major identified challenges to the commercialization of innovative sustainable solutions in the food packaging area will be highlighted, focusing on “full bio-packaging” solutions, which means issues from non-food renewable resources which are biodegradable in natural condition. A special focus will be paid in this part on the need of *early guidance tools* for packaging users and producers to efficiently choose the suitable packaging material and *fast track* innovations up to market penetration. Then, in a third part, some solutions to overcome those problems will be presented based on last inputs from state of the art that bring significant advances in the field of eco-innovative packaging solutions in terms of knowledge, technical up-scaling, user-driven approaches and decision-support tools to provide *new products and services*. This will be illustrated by using a thorough analysis of the scientific literature, paying attention also to the key elements of the European environmental and safety regulation on this topic. Lastly, in a fourth part, the expected impact by horizon 2050 of the aforementioned solutions will be summed up.

## Primary Fundamental Role of Food Packaging

The primary fundamental role of food packaging is to preserve food quality and safety, to reduce food waste and food-borne diseases, and to reduce the corresponding useless negative impact that producing and distributing uneaten or inedible food has on our environment and economy. That means that packaging functional properties must fit the food requirements, especially its mass transfer properties.

Mass transfers through the packaging material (transfer of gases, water vapor, aroma compounds, etc.) play a major role in the control of food degradation reactions by defining around the product an atmosphere whose composition is favorable to the slowing down of the reactions, thereby extending food shelf life. For instance, the control of O_2_ concentration in headspace limits oxidation reactions and growth of aerobic microorganisms, two main causes of food deterioration during storage. This technology, called Modified Atmosphere Packaging (MAP), relies on the modification of the internal atmosphere by the product itself (passive MAP) or by gas flushing or use of gas emitters or scavengers (active MAP) (8, 19, 20). In both cases, the optimal atmosphere is achieved thanks to the mass transfer properties of the packaging material, especially its permeability toward gas and vapors, i.e. its ability to let migrants pass from the external atmosphere toward the internal one.

Permeability properties of the food packaging, also called barrier properties, rarely fully meet the food requirements. These barrier properties are either too low (case of O_2_ sensitive food products for which high barrier materials are required) or too high. We can cite for example, the case of respiring products such as fresh fruits and vegetables, where the plastic film is perforated to compensate for the too high barrier properties of the packaging.

**Figure 1:**
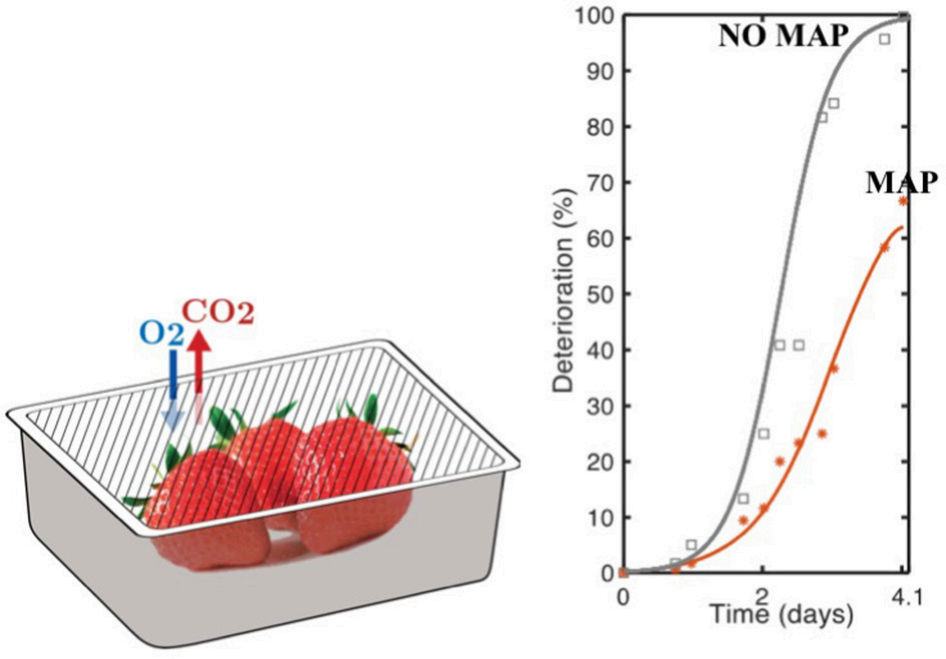
Benefit of MAP (Modified Atmosphere Packaging) compare to a control with NO MAP to limit the degradation rate of strawberries “*Charlotte*” variety (graph on the right)-adapted from Matar et al. (10).

As a result, current packaging is usually over or poorly designed and not well adapted to the food needs. Packaging does not efficiently and sufficiently contribute to maintain food quality although much higher benefits in terms of reduction of food losses could be achieved using well dimensioned packaging material (Figure 1) (9, 10, 19).

Packaging is usually wrongly considered as an additional economic and environmental cost rather than an added value for food loss reduction by improving food shelf-life. In order to contribute to solving the environmental issues of the food/packaging system as a whole, it is necessary to consider, in addition to the environmental impact of the packaging material itself, its contribution to the reduction of environmental impact of food loss and waste (8, 12).

## Current Challenges in the Field of Food Packaging and Sustainability

In the very dynamic worldwide food packaging sector, marketed innovations essentially focus on practical and easy-to-use aspects as well as conviviality and aesthetics for consumer attractiveness. Some of the marketed innovations are claiming to be sustainable either by their resources (bio-based) or their end of life (biodegradable) but without a full and fair assessment of their overall environmental benefit. Most of these eco-friendly innovations are less eco-friendly than expected: for instance, materials vary significantly in terms of quantity of renewable resources used in their formulation and may or may not be readily compostable as is often claimed. None of these innovations claimed to be sustainable for its usage benefit, which is food loss reduction.

The crucial societal stake of sustainable food consumption still needs to be bridged with a wealthy R&D sector, proposing a large reservoir of innovative packaging technologies that will improve packed food sustainability.

In particular, a lot of research has been done on the development of **bio-packaging** solutions, i.e., either bio-based packaging materials made from renewable resources and/or biodegradable materials. However, packaging stakeholders are facing the difficulty of overcoming specific **technical issues** with these bio-packaging materials that currently hinder large market uptake. These technical issues are in particular, an avoidable raw material variability and a too narrow processing window, compared to common oil-based counterparts, that hinders their scaling up and diffusion among packaging producers. In addition, the **lack of tools** to help users to tailor packaging to food needs (e.g., to fit packaging mass transfer properties to food requirements) and to decipher the real sustainability of bio-packaging innovations and packaging at large, especially in terms of food losses reduction, prevents stakeholders to fully seize the economic, societal, and environmental opportunities of these innovations.

### The Confusing Long Term Environmental Benefit of Eco-Friendly Packaging Solutions

Despite extremely dynamic research and development on bio-sourced and/or biodegradable materials (more than 1,400 scientific publications/year on the last 10 years–Table 1), commercially available bio-packaging does not yet properly meet the huge market and consumers demands.

**Table 1:** Summary of Tensile Properties (Tensile Strength, Tensile Modulus, and Strain at Break) and oxygen permeability of some Biodegradable Polymer Matrices [adapted from (21)].

**Polymer**	**Tensile strength (MPa)**	**Young's modulus (GPa)**	**Strain at break (%)**	**PO2 x 10^17^ (mol m^−1^ s^−1^ Pa^−1^)^a^**
PCL	19 à 21	0.21 à 0.33	300 à 897	26
PBAT	>84	0.04	>200	–
PBSA (Bionolle)	20	0.44	20	–
PLA	21	0.35	3	41
P(HB-co-HV) with 3% HV	40	3.5	5	1–7
P(HB-co-HV) with 3% HV and 20% of milled wheat straw^b^	19.6	3.03	1.03	–
Polypropylene	34.5^c^	1.7^c^	400^c^
Polyethylene -terephtalate	56^c^	2.2^c^	–	0.72^d^
LDPE	10^c^	0.2^c^	620^c^	95.7^e^

a *Measured at ambient temperature and 0% RH*.

b *From Berthet et al. (22)*.

c *From Khanna and Srivastava (23)*.

d *From Auras et al. (24)*.

e *From (10)*.

Bio-packaging development is hampered by serious controversies about its technical, social, and environmental benefit (ambiguous claims on environmental impacts, competition between food and non-food usage of agricultural resources, high environmental cost of already existing “bio” solutions, troublesome compostability of PLA, green washing suspicion, and more) (17).

The “bio” label itself (bio-based, biodegradable, bioplastic…) is misunderstood by customers. While they might interpret the “biodegradable” labeling to mean “fit for home composting,” in reality, the large majority of current biodegradable plastics (e.g., PLA) can only biodegrade under very specific conditions of constantly high temperature and humidity in industrial composting installations, and they are neither fit for home composting nor do they decompose in reasonable time when littered, implying damaging consequences for fauna and flora (e.g., aquatic ones) (15).

Encouraged by a favorable European regulation [EU Circular Economy Package, EU Waste legislation (25), etc.], recent innovative research has focused on developing bio-plastics from organic waste streams (crop residues, agro-food by-products, sewage sludge, etc.) seeking to enter a circular economy concept that does not compete with food usage and that is fully biodegradable to respond to the overwhelming negative externalities of our plastic packaging: today, 32% of plastic leaks out of collection systems into the environment, of which 8 million tons leak into the ocean each year. The latter is equivalent to dumping the contents of one garbage truck into the ocean every minute, which is estimated to increase to the contents of four trucks per minute by 2050 if no action is taken (13).

There is a real need to develop convincing sustainable packaging materials decoupled from fossil feedstocks, with no competition with food resources and with a real advantage to solve the issue of the accumulation of persistent plastics in our environment. This could be achieved by enhancing the conversion of *agricultural and agro-food residues* into “*naturally biodegradable”* packaging^3^ with a fair and transparent eco-efficiency performance assessment. It is also necessary to enlarge industrial process-ability and functionalities of these materials that must be tailored to usage requirements while optimizing their cost. The organic residues used as feedstocks for this bio-packaging production must be unavoidable and worthless by-products and residues of agricultural and agro-food industries that are thus turned into value-added raw materials for bioplastics production^4^.

### Need to Clearly Assess the Benefits of Packaging Solutions to Reduce Food Waste and Losses

Although there is plenty of evidence of the benefits of using innovative packaging solutions to extend food shelf-life, there is no general approach that permits to assess the shelf life of a packed product and especially the gain of shelf life that could be achieved by using well designed primary packaging, with functional properties that match the food requirements well. For instance, searching on the Easy Web of Science tool for the last 10 years, with the following keywords: Modified Atmosphere Packaging, and Shelf life separating those two keywords with the connector AND, 1,566 articles were found (done in May 2018) proving the dynamism of research in that field.

Among innovative packaging solutions, MAP and especially active MAP, where active compounds are, for instance, emitted from packaging toward headspace creating a modified atmosphere that limits microbial spoilage, is a good example of eco-packaging solutions (19, 27). However, these solutions remain difficult to adapt and up-scale because they need to be clearly fitted to the specific needs of the food. For instance, in the case of passive MAP when the product itself creates the modified atmosphere due to its aerobic metabolism (e.g., the case of respiring product), the O_2_ and CO_2_ permeability property of the film must be adapted to the respiration rate of the product (11, 28). There is a high risk of failure if empirical trial-and-error approach is used to adjust the film permeability, the gaseous atmosphere composition or the quantity of active compound to obtain the expected effect on food quality and safety preservation (19, 29, 30). Currently, no food requirement driven approach is commercially used or available to help industry to use active packaging.

Moreover, regulatory constraints regarding solutions that imply solutes or volatiles migration (such technologies have to comply with both food and packaging regulation) create additional cost and delay before market uptake. In addition, there is a general consumers' widespread suspicion on sachets and emitters due to their possible interaction with the food product and misunderstanding of their role.

As regards the usage benefit, the reduction of food waste and losses achieved by using well-dimensioned packaging solutions, especially active packaging solutions, still need to be quantified and disseminated to all stakeholders in an informative and easy-to-understand manner, especially to consumers in order to increase their awareness and acceptance of such packaging as sustainable food packaging solutions.

Full assessment of environmental- and socio-economic benefits of *packaging solutions* is not straightforward. There is an urgent need of a holistic approach to tailor packaging materials, validate their usage benefit and increase end-users' acceptability. This could be achieved by (1) setting up a requirement-driven approach to globally deal with issues related to efficacy assessment, compliance with food and food contact material regulation, environmental constraints and consumer's acceptance and (2) driving a concerted and collaborative initiative including all relevant stakeholders (packaging producers, food companies, retailers, consumers) in the early stage of the deployment and validation of usage benefit of packaging solutions for increasing perceived benefits and awareness by all citizens.

The *high fragmentation* of today's innovation strategy in the packaging sector does not enable stakeholders to seize all opportunities for new food packaging solutions. There is an obvious lack of concentration between the numerous and diverse stakeholders throughout the whole packaging material life cycle, from the producers, food manufacturers to the waste managers. Particularly, the full assessment of the environmental benefit of eco-innovative solutions in terms of material (resources and waste) and usage (reduction of food waste and losses) is currently not achieved.

The adoption of eco-innovative packaging solutions by SMEs, that represent more than 90% of the EU food and packaging sector, is currently hampered by the fact that the large majority of these SMEs do not have a dedicated packaging manager and decision makers often lack the background knowledge, tools and network contacts regarding packaging issues that would otherwise enable them to move forward. To ensure competitiveness of EU SMEs, it is necessary to provide them with tools and reasoning that will enable them to enter and dominate this specific market of sustainable food packaging solutions where packaging solutions must be tailored to fit food and market specificities.

There is an urgent need to develop *early guidance tools* for packaging users and producers that will help them to *fast track* sustainable innovations up to market penetration. Based on *user-driven strategy* able to fit packaging to foods and market diversity, complexity and requirements, these decision-supporting approaches and tools should be able to design and communicate, in a user-friendly format, eco-innovative packaging alternatives by setting up, for instance, scores of sustainability performance. These calculated indicators could be a basis for the setting up of front-of-package sustainability labels, to be further disseminated to all end-users, especially consumers.

## Solutions and Tools to Align With the Principles of Circular Economy for Food Packaging

To address the main challenges listed above, there are some solutions, which are all underpinned by and aligns with principles of the circular bio-economy. Most of them are still in their infancy and some efforts are still needed to market them and enable the food packaging economy to create virtuous cycles instead of depletive ones and harness the whole innovation potential of research made in the field of food, material, environmental, and computer sciences.

In the following, the most promising solution in the development of bio-packaging solutions issued from the conversion of agro-food residues is presented. Then most recent developments, at the crossroads of food engineering and computer science, that allow to tailor packaging to food needs and to help users to select sustainable packaging solutions, are presented.

### Converting Agro-Food Residues Into Innovative Bio-Packaging Solutions

The demand for bio-packaging solutions is growing worldwide. For instance, the European market for bio-based polymers (biodegradable or not) represents a current market value of almost € 4.5 billion, representing a CAGR (Compound Annual Growth Rate) of 21% and is estimated to increase to 2 M tons by 2020 (31). But this market remains very small with only 2% of the total polymer market. Among bio-based polymers, biodegradable polymer-based packaging represents only 0.8 M tons, € 2 billion (2016) (32). The main barrier to market uptake is attributed to technical bottlenecks related to the functional and production specificities of bio-based materials that are quite different from petrochemical plastics.

**Figure 2:**
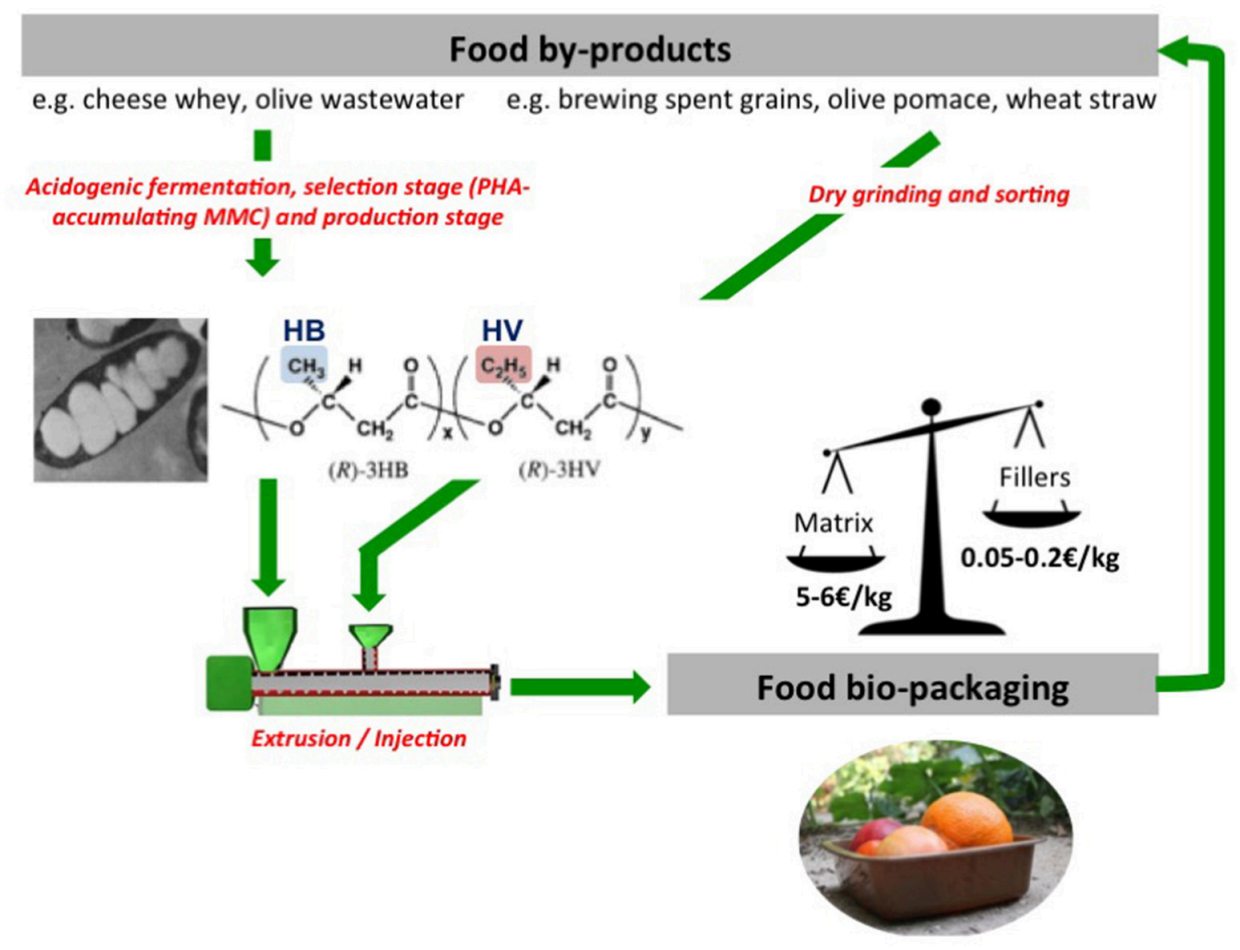
Microbial engineered polymers (example of PHA) permit conversion of food by-products into food bio-packaging (EcoBioCAP FP7 2011–2015).

With the objective to convert agricultural and agro-food residues into “naturally biodegradable” packaging, microbial (bio-polyesters) engineered polymers enable a real environmental, economic and industrial added value by adopting regenerative process-oriented systems adapted to conventional and local industries. Among biodegradable microbial polymers, polyhydroxyalkanoates (PHA) and particularly the copolymer polyhydroxy (butyrate-co-valerate), P(HB-co-HV), are considered among the most promising substitutes of oil-based synthetic polymers (33–36) to tackle current negative externalities of our plastic packaging-more than 70% of accumulation of persistent plastic in the environment through landfilling and leakage (13). Among their advantages, they can be biologically synthesized using various feedstocks such as agro-food and urban by-products, residues and wastes, either liquid or solid. They are completely biodegradable in both natural (soil) and marine conditions, in contrast to other commercially available bioplastics (PLA, PCL, etc.) and a large number of copolymers displaying different functionalities can be produced by controlling the feedstocks and the microorganisms. However, currently available commercial grades [either P(HB-co-HV) or PHB] are still synthesized from noble food resources^5^ using pure cultures of particular microorganisms (GMO origin) contributing to a prohibitive market price (about 5 €/kg) as compared to the one of conventional plastics. In addition, they display a limited range of hydroxyvalerate (HV) content (max. 3 wt%) that hinders their suitability for food packaging application due to high thermal sensitivity, low viscosity at the melting state, and low crystallization rate (37, 38). The FP7 EcoBioCAP project^6^ demonstrated the feasibility, using food industry by-products as feedstock (olive wastewater or cheese whey) and mixed natural microbial cultures (MMC), of lab scale production of a P(HB-co-HV) with a HV fraction (in the range of 10–25%) higher than the current commercial grade (39–41). This higher HV fraction induces some polymer structural changes that can be advantageous to its processing and conversion into packaging (42, 43). Higher HV contents could be achieved using Volatile Fatty Acids (VFAs precursors) with a high propionic acid content. The incorporation of low cost lignocellulosic fillers stemming from lignocellulosic solid residues into P(HB-co-HV) permitted to tailor functional properties, especially water vapor and oxygen permeability, while decreasing the overall cost of the final bio-composite packaging material and maintaining its biodegradability (21, 22, 44, 45) (Figure 2). Incorporation of lignocellulosic fillers tends to decrease the ultimate tensile properties because of a lack of adhesion between the hydrophobic matrix and hydrophilic fibers (22). Globally, the mechanical properties are governed by that of the PHBV matrix which is, for the commercial grade with low HV content, too brittle to be used for flexible packaging application (Table 1).

To go further in the industrial deployment of PHA-based material, PHA conversion must be scaled-up based on the use of an optimized eco-efficient mixed microbial culture (MMC) based process. This allows to decrease investments and operating costs of PHA conversion with respect to pure culture and is made easier by using non-costly by products such as feedstock (35, 39). This type of process will enable the bioconversion of agro-food residues (no competition with food usage) into value-added material that is a better alternative use for bio-waste rather than only energy or compost. Municipal bio-waste could also be converted into PHA as is currently being explored in the framework of the RES-URBIS H2020 project^7^ To enlarge P(HB-co-HV) industrial process ability and make it compatible with conventional packaging processing techniques, the HV content of the synthesized polymer must be controlled in a wide range using a combination of customized feedstock pre-treatment (acidogenic fermentation performed in conditions that trigger production of propionic acid in the VFAs mixture) and VFAs bioconversion into PHA. In addition, the combining of synthesized PHAs with low cost ligno-cellulosic fibers into bio-composites should continue to be explored to tailor cost and functionalities of PHA-based materials to food usage requirements as well as mechanical, transport, and cost properties.

### Tailoring Packaging Properties to Reduce Food Waste and Losses

Packaging is a particular key player to improve food preservation, quality and safety conditions, and thus reduce food losses through, notably, setting up of Modified Atmosphere Packaging (MAP) technologies. In MAP, one of the main roles assigned to packaging materials is the control of mass transfer between the food, the packaging, and the atmosphere, i.e. ,permeation of gases from the surrounding atmospheres, absorption of these same gases (e.g., O_2_ scavengers) or diffusion of active molecules voluntarily added in the packaging material (anti-microbial emitters).

MAP design is complex and requires knowledge on packaging material, food characteristics, and optimal gases composition and is thus dependent on the product (10, 11, 46). In the case of passive MAP, Tailorpack (http://plasticnet.grignon.inra.fr/IateTools/TailorPack) is an example of a user-friendly software able to design packaging for fresh produce such as fruit and vegetables. A mass balance of gases composition in the headspace is done by taking into account the permeation of the gases through the film via Fick's first law and the respiration of the fruit modeled using Michaelis and Menten's law (10, 28, 47). For MAP of non-respiring fresh products (e.g., meat, ready-to-eat food products, etc.) similar tools exist that help the user to choose the suitable packaging material and atmosphere composition to limit growth of pathogens (48, 49). However, these tools are limited to some specific food applications.

**Figure 3:**
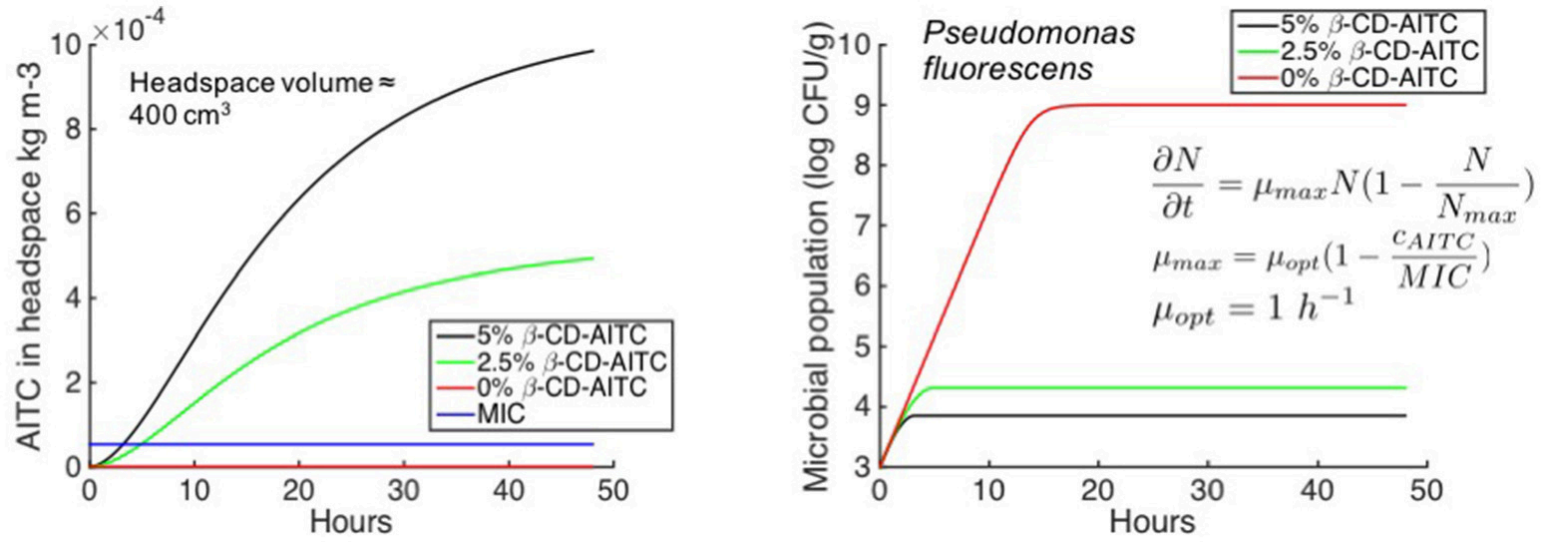
Prediction of the AITC active compound release (on the left) toward headspace as a function of quantity of active compound added in the formulation of the packaging and correlated predicted effect on the growth of *Pseudomonas fluorescens* (on the right) [adapted from (51)].

Among the latest developments in antimicrobial emitters, a promising way to develop indirect contact anti-microbial packaging is the use of volatile compounds, encapsulated in RH-sensitive macromolecules that prevent release during storage in dry conditions. Once exposed to moisture, release of the molecule is triggered and then diffuses into the headspace toward the food surface where microbial growth usually takes place (50, 51). Although widely available on the Asian market (see for example the AITC-based Wasaouro^TM^ film^8^, they are almost inexistent on the EU market (27, 52, 53) because of more restrictive EU regulatory requirements (54) and difficult efficacy optimization (55) principally due to the complexity of the RH-triggered release mechanism. Among volatiles, organic aroma compounds from essential oil extracts such as allyl isothiocyanate (AITC) from mustard or carvacrol from oregano, have been proved to be particularly efficient on main microorganisms (56–58) at doses that are below the detection threshold by sensory panel. Recently, setting up dedicated mathematical algorithms that predict the complex diffusion-reaction system and kinetic release toward headspace, Kurek et al. (51) tailored active biodegradable material in such a way that it complies with the food requirements (Figure 3).

The next step of a requirement driven approach to design packaging materials will be to consider, at the early stage of their scaling up, all the food, consumer, market, and legal requirements that the material should fulfill. In the specific case of active packaging, when volatiles are emitted toward the food, consumer exposure, including all sources of the substance of concern such as natural occurrence in food products must be considered in addition to food needs in terms of quality and safety preservation and shelf-life extension. Indeed, for active packaging solutions to be commercially viable and successfully adopted by the market, it is necessary to ensure that they meet the regulatory requirements while ensuring intended efficacy and limited impact on food sensory properties (especially for volatiles also used as flavorings). In particular, to validate the fact that active materials have, in operational conditions, a final beneficial outcome in terms of usage benefit that outweighs the possible extra expenses of adding the new technology, it is necessary to demonstrate their positive role to decrease food waste and thus contribute to increase sustainability of the food packaging system as a whole.

### Early Guidance Tool to Develop and Select Sustainable Packaging Solutions

For almost a decade now, Europe has been investing a lot in research for new developments in packaging technologies and was perceived as a powerful market with an immense potential demand^9^. All forecasts showed a dramatic growth in production, use and acceptance of bio-, and smart packaging technologies for 2000–2010, but these figures have proved to be very optimistic. Even though several new technologies were successfully developed at lab-scale all around Europe (more than 15,000 scientific papers dealing with bio-, and active technologies were published on the 2010–2015 period, Table 2), they never or very rarely reached the market (>500 exploited patents over the same period). Many factors have contributed to this failure including the resistance of the food industry and consumers to adopt unknown technologies, the costs of the new implementation, the inefficiency and lack of competitiveness of the new technologies and regulatory barriers. But the biggest challenge remains the lack of collaboration and exchange between stakeholders of the food chain (R&D centers, food and packaging manufacturers, legislators, consumers) resulting in lab-scale prototypes that, though efficient, never meet market expectations in their entirety, in terms of potential applications, added-value, risk-benefit balance, compliance with EU rules or consumer trust.

**Table 2:** Overview of the current innovation status in the sustainable food packaging sector.

**Period: 2010–2015**	**Active AND packaging**	**Biopolymers AND bio-based AND bioplastics**
Nb of scientific publications^*^	8,250 (900 in 2015)	11,000 (1,400 in 2015)
Nb of patents^**^	89 (11 in 2015)	754 (26 in 2015)
Nb of exploited patents^***^	53 (6 in 2015)	452 (15 in 2015)
Current deployment ratio^****^	1%	4%

* *From the Web of Science*.

** *Worldwide database/Espacenet*.

*** *calculated from the paper of Giuri et al. (59) that claims that about 40% of patents are not used taking all sectors into consideration*.

**** *Ratio of exploited patents on papers*.

*Something missing in table above—Active AND …… packaging*.

The efficiency of new packaging solutions to reduce the overall environmental impact of the food/packaging system is never assessed on large-scale market nor communicated in easy-to-understand format to end-users. Thus, almost 50% of the food and packaging industries specialists are not fully aware of new available technologies (60). The situation is similar for consumers: they are generally not aware and are generally skeptical regarding new technologies that they do not fully understand, especially active packaging (e.g., Actipak final report) (61–63).

**Figure 4:**
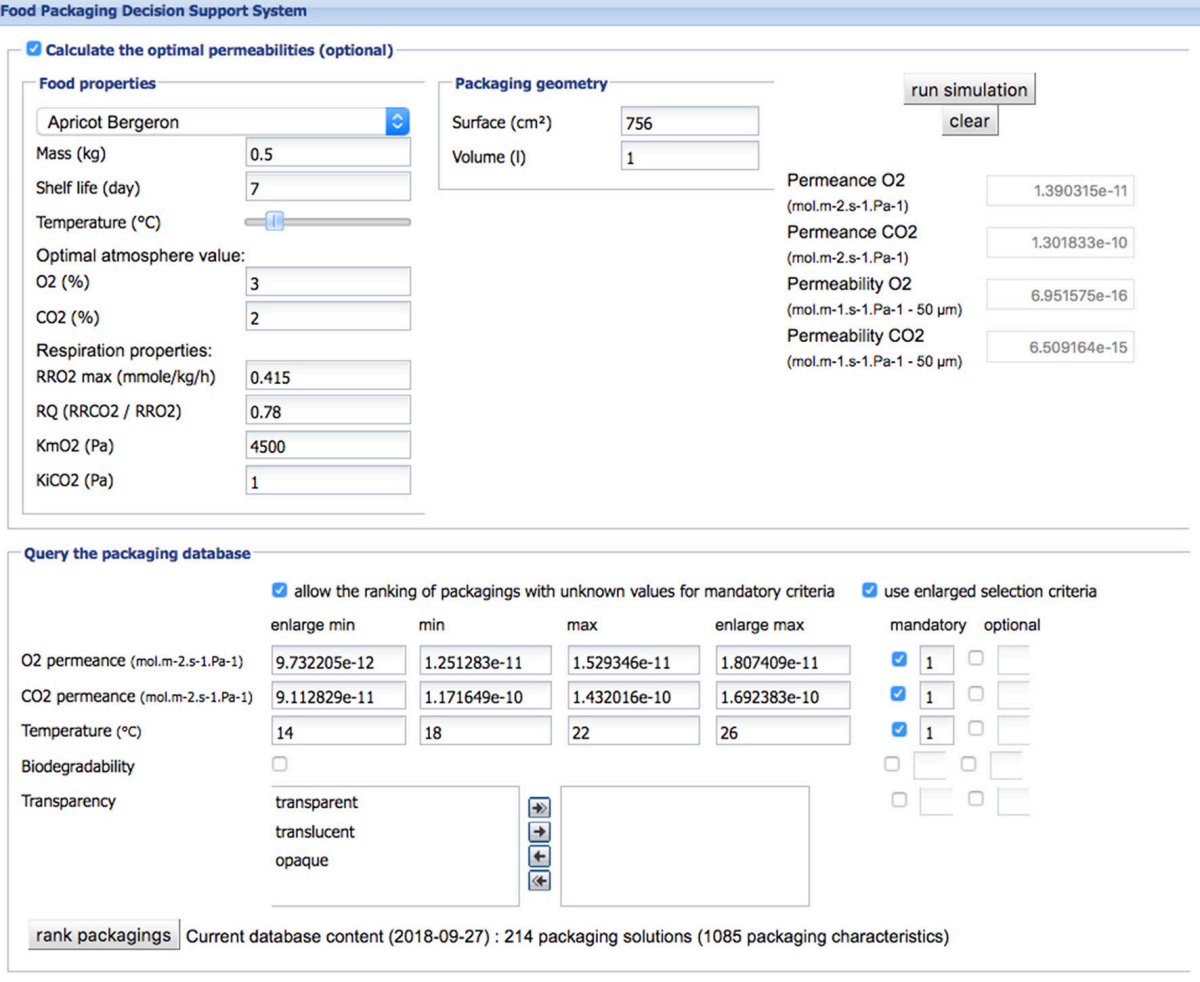
Main window of the DSS EcoBioCAp with indication of the values of permeances calculated for the case study Apricot and building of the multi-criteria query.

**Figure 5:**
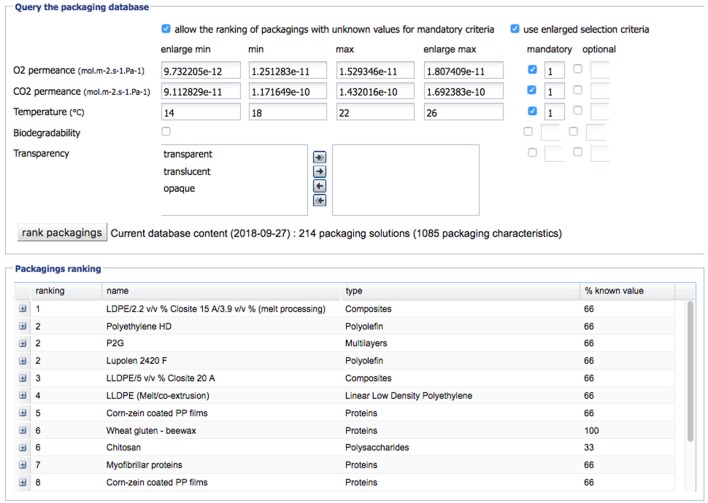
Ranking of the most suitable packaging solutions proposed by the DSS EcoBioCAP for the case study “Apricot”.

Moreover, the food and packaging industries encompass a large number of SME's, which face specific difficulties through not having sufficient in-house technical resources and needing to rely on suppliers for advice. Fully efficient advice resulting in a direct implementation of new technology in SMEs is rarely available as expertise on packaging innovations is fragmented, based on a lot of, multi-disciplinary knowledge owned by many different actors (raw material suppliers, food manufacturers, distributors, researchers). As a result, SME's may not always be using the best and most sustainable food packaging solution. In the framework of the FP7 EcoBioCAP project, the first lab prototype of a multi-criteria decision software for modified atmosphere packaging of respiring fruits and vegetable has been developed together with an argumentation-based tool for management of conflicting viewpoints between preferences expressed by the involved parties (64–67). They help to handle the complex decision in the field of packaging choice and design considering only a restricted range of criteria at the moment (Figures 4, 5).

By proposing in depth information about eco-innovative packaging technologies such as value-added, consumer acceptance, sustainability performance, up-scaling ability, etc. the next generation of Decision Support System (DSS) should provide unique and specific guidance to food and packaging SMEs in terms of technical assistance for the selection among eco-innovative packaging alternatives. It is a necessary evolve to a wider acceptance and assurance that these organizations will remain competitive. To the best of our knowledge, this tool does not exist yet in the food packaging sector.

## Which Impacts Should be Expected by 2050?

The next generation of food packaging should significantly contribute to reduced waste in both food and packaging materials, and its negative impacts on the environment (e.g., resource utilization, greenhouse gas emissions, pollution) by 2050.

Indeed, the carbon footprint of food produced and not eaten (around 100 million tons annually in the EU) is estimated to be equivalent to 495 million tons of CO_2_. Globally, the blue water footprint (i.e., the consumption of surface and groundwater resources) of EU food wastage is about 37 km^3^, half the volume of Lake Geneva. Produced but uneaten food occupies almost 210 million hectares of land. Modeling suggests that, if nothing is done, food waste could rise to over 200 million tons by 2050 (68, 69).

In the meantime, 23 million tons of plastic packaging are produced each year at European level (92 million tons expected in 2050). If production and use continue within the current linear framework, worldwide, by 2050 the plastic industry will represent 1,124 million tons of plastic materials, 20% of total oil consumption, 15% of carbon budget^10^ and if nothing is done there may be more plastic than fish in the ocean, by weight (13).

By promoting market uptake of packaging innovations enabling extension and better management of food shelf-life, 50% decrease of food waste at the retail and consumer level could be expected by 2050, i.e., saving about 100 million tons of food which corresponds to an absolute decrease of 250 million tons of CO_2_-equivalent, about 18 km^3^ of water resources and 100 million hectares of land recovered (70). This achievement is in line with the EU targets^11^

If one in two food packs are made of a “bio-benign” material by 2050, 50% of packaging waste reduction could be achieved, i.e., about 46 million tons of plastic waste less, reducing the negative impacts of plastic accumulation in natural systems and the long term adverse effects expected.

By substituting one pack out of two with organic waste-based packaging, net saving of about 43 MTOE of virgin oil based resources is expected on average by 2050 at European level and more than 150 MTOE on a global level. These savings represent an absolute reduction of GHG emissions of 120 million tons of CO_2_-eq^12^ at EU level and 500 million tons of CO_2_-eq worldwide (direct CO_2_ emissions only).

In summary, at European level, expected reduction on both food and packaging waste, thanks to sustainable food packaging solutions, would correspond to a net reduction of 370 million tons of CO_2_-eq, representing a net saving of about 10% of GHG emission according to 2050 EU objective to be consistent with the 2°C limit (IEA 450 scenario, EEA greenhouse gas–data viewer).

The next generation of food packaging will support the transition from a linear to a circular economy.

**Figure 6:**
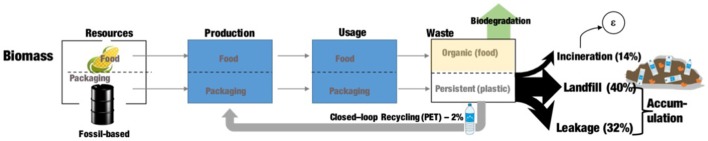
Current linear status of today's food packaging economy [data from (13)].

Our current plastic-based food packaging economy is an iconic linear application (Figure 6): from the 78 millions of tons of plastic packaging produced each year at European level, 98% originates from virgin oil-based feedstock, and after-use, only 14% is recycled, far below the global recycling rates for paper (58%)^13^and iron and steel (70–90%) (71). Forty percent of plastic packaging is still put in landfill and more than 30% leaks into natural systems (especially oceans). If the current strong growth of plastics usage continues as expected, the consumption of oil resources by the entire plastics sector will account for 20% of the total oil consumption by 2050 (13). Currently 8 million tons of plastics leak into the ocean each year worldwide for a total amount of 150 million tons of plastic waste in the ocean (14), 62% of it is packaging.

If recycling has been seen as essential to the setting up of an effective after-use plastics economy, safety and environmental issues of closed-loop recycling^14^ (e.g., bottle-to-bottle for PET) and lack of resilient secondary markets for cascaded recycling^15^(recycling of plastics into other applications than food packaging) level off its development to the current low level. On the whole almost half of PET is not collected for recycling, and only 7% is recycled bottle-to-bottle (72).

The thermo-mechanical recycling as is currently applied in bottle-to-bottle technologies, entails a deterioration of the material properties by damaging or shortening the polymer chains of the PET and the presence of contaminants and impurities from pre-use and degradation products of monomers and additives, resulting in a down-cycling^16^of the material (73). The safety of recycled plastics for food contact, by nature, needs the recovery of virgin material that could not be achieved with low environmental cost using current methodologies (74, 75). Recycling is not the unique solution to be deployed to solve the plastic economy issue. Alternative packaging solutions must be deployed.

**Figure 7:**
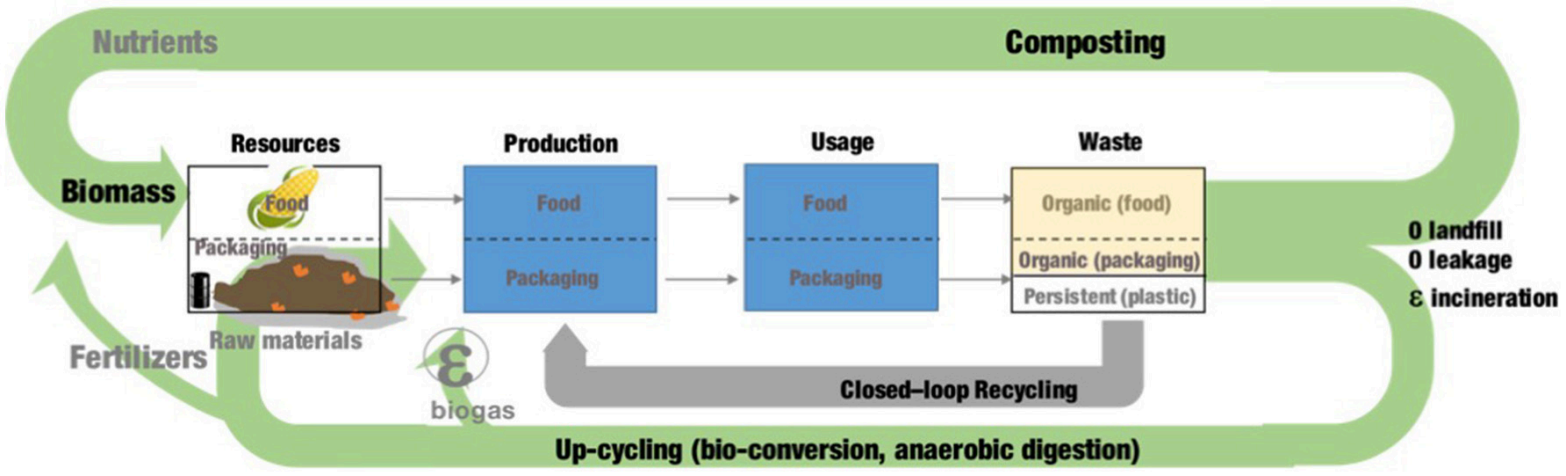
Unlocking the circular economy potential of the food packaging chain, a prospect for the future.

Aligned with circular economy principles, by converting the unavoidable part of organic wastes into new materials (100% biodegradable bioplastics), the next generation of bio-waste based materials will create an innovative, more resilient and productive waste-based food packaging economy by decoupling the food packaging industry from fossil feedstocks and permitting nutrients to return to the soil (Figure 7).

More especially, we can imagine by 2050, being able to produce 50% of the European food packaging materials from renewable, non-food resources by using up-cycling of organic (food and packaging) wastes, the other 50% oil-based materials being closed-loop recycled. This bio-based packaging (about 46 MT by 2050) will be fully biodegradable and home-compostable (100 million tons of organic food and packaging waste could be converted into up to 50 million tons of compost) solving current issues of persistent plastic waste accumulation in line with EU Circular Economy Strategy (e.g., banning of landfilling by 2050).

In the meantime, the use of these organic residues in an up-cycling loop through bio-conversion processes (aerobic accumulation, anaerobic digestion, etc.) will allow to produce new materials (bioplastics), energy (to be reused for the food and packaging production steps), and ultimately some fertilizers (76).

By shifting our current food & packaging industry to a circular economy development path would generate annual total benefits of up to € 0.6 trillion in Europe (estimated from data given in Growth Within: A Circular Economy Vision for Competitive Europe (77).

## Author Contributions

All authors contributed to the conception and design of the present review. PB supervised the decision support tool presented in Figures 4, 5 with associated database. SG performed the mathematical modeling part. VG wrote the first draft of the manuscript. HA-C, NG, SG, VG, PB, and CF wrote sections of the manuscript. All authors contributed to manuscript revision, read and approved the submitted version.

### Conflict of Interest Statement

CF was employed by the company Coopbox. The remaining authors declare that the research was conducted in the absence of any commercial or financial relationships that could be construed as a potential conflict of interest.

## Footnotes

^1^The retail or consumer pack that contains the sales unit (e.g., a plastic bag, glass jar, or steel can, or a plastic crate for loose fresh produce).

^2^PlasticsEurope, Plastics—the Facts 2013 (2013); PlasticsEurope, Plastics—the Facts 2015 (2015).

^3^**Naturally biodegradable**: fully biodegradable in natural land conditions or home composting conditions as opposed to industrially compostable materials such as PLA.

^4^**Agro-waste** is defined as plant or animal residues that are not (nor further processed into) food or feed, and create additional environmental and economic issues in the farming and primary processing sectors. These residues should not be mistaken with the avoidable agro-food waste. Unavoidable primary agricultural residues account for about 50% of the fresh weight of harvested crops and represent a potential of 90 MTOE, far ahead of other waste sources such as round wood production (57 MTOE), municipal and other waste (42 MTOE) and tertiary forest residues (32 MTOE) (26).

^5^see for example the most widely available production of the Chinese company, Tianan synthesized from glucose syrup.

^6^EcoBioCAP FP7 (2011–2015) ECOefficient BIOdegradable Composite Advanced Packaging (2011–2015).

^7^RES-URBIS H2020 (2017–2019) REsources from URban BIo-waSte.

^8^Mitsubishi-Kagaku Foods Corporation. Wasaouro^TM^ products. http://www.mfc.co.jp/wasaouro/e/products/

^9^Pira International, 2009; Research And Markets, 2010. The Freedonia Group, 2011.

^10^Energy used in production and carbon released through incineration and/or energy recovery after-use (i.e., 20% in 2050). Carbon budget based on 2 degree scenario.

^11^http://www.un.org/sustainabledevelopment/sustainable-consumption-production/

^12^1 toe 11630 kWh and 0.24 kg CO_2_/kWh.

^13^International Council of Forest and Paper Associations, Statement on Paper Recycling (2014).

^14^Recycling of plastics into the same or similar-quality applications.

^15^Recycling of plastics into other, lower value applications.

^16^Converting waste materials into new materials or products of lesser quality and reduced functionality.
